# Banknotes as a Source of Drug and Pharmaceutical Contamination of the Population

**DOI:** 10.3390/toxics13040242

**Published:** 2025-03-24

**Authors:** Nina Petrovičová, Jarmila Látalová, Paula Bimová, Anna Krivjanská, Veronika Svitková, Ján Híveš, Miroslav Gál, Miroslav Fehér, Andrea Vojs Staňová, Alexandra Tulipánová, Alexandra Paulína Drdanová, Jozef Ryba, Zuzana Imreová, Peter Nemeček, Barbora Jančiová, Tomáš Mackuľak

**Affiliations:** 1 Department of Environmental Engineering, Faculty of Chemical and Food Technology, Slovak University of Technology in Bratislava, Radlinského 9, 812 37 Bratislava, Slovakia; 2 Department of Inorganic Technology, Faculty of Chemical and Food Technology, Slovak University of Technology in Bratislava, Radlinského 9, 812 37 Bratislava, Slovakiaveronika.svitkova@stuba.sk (V.S.);; 3 MicroPoll s.r.o., Vazovova 5, 812 43 Bratislava, Slovakia; 4 South Bohemian Research Center of Aquaculture and Biodiversity of Hydrocenoses, Faculty of Fisheries and Protection of Waters, University of South Bohemia in České Budejovice, Zátiší 728/II, 389 25 Vodňany, Czech Republic; 5 Department of Analytical Chemistry, Faculty of Natural Sciences, Comenius University in Bratislava, Mlynská Dolina, Ilkovičova 6, 842 15 Bratislava, Slovakia; 6 Institute of Chemistry and Environmental Sciences, Faculty of Natural Sciences, University of Ss. Cyril and Methodius in Trnava, Nám. J. Herdu 2, 917 01 Trnava, Slovakia; peter.nemecek@ucm.sk (P.N.); barbora.janciova@ucm.sk (B.J.)

**Keywords:** pharmaceuticals and illicit drugs, LC-MS/MS analysis, occupational exposure, cashiers, banknote contamination, environmental micropollutants, wastewater pollution, surface transfer

## Abstract

This study investigates the potential contamination of cash register employees in the Slovak Republic with 148 selected pharmaceuticals, illicit drugs, and their metabolites. Of these, 42 substances were detected, and it was found that the target group—cashiers—regularly handle large volumes of banknotes, increasing their exposure to contaminants compared to the general population. This study revealed that commonly prescribed and over-the-counter medications significantly contribute to the contamination of cash registers. This study found that cashiers exhibited notably higher detection rates of antibiotics, including penicillin-V (2×), azithromycin (23×), and erythromycin (up to 64×), than the general population. Additionally, there was an alarming increase in illegal substances, with methamphetamine levels rising fivefold and cocaine up to fifteenfold. This study highlights a broader environmental concern, suggesting that routine handling of contaminated banknotes may lead to the transfer of micropollutants. Furthermore, personal hygiene practices, particularly handwashing, could play a role in introducing pharmaceuticals and narcotics into wastewater, potentially contributing several milligrams of contaminants daily.

## 1. Introduction

Presently, research is concentrated on the presence of micropollutants like pharmaceuticals or drugs in different environmental compartments [1,2,3,4,5,6,7,8,9]. Expert studies provide information on the distribution of both illegal and legal drugs and their metabolites in wastewater, surface water, and drinking water [1,2,3,4]. Analyzing the quality of waters, particularly wastewater, serves as a foundational method to gather data on the consumption patterns of pharmacological products. Moreover, such examinations offer valuable insights into the potential consumption or production of illegal drugs, presenting a significant interest from this perspective [4,5,6,7]. The analysis of drug and medicine traces on banknotes has been explored in various studies, highlighting the intersection of public health and forensic science in addressing substance abuse and monitoring drug circulation within communities. This approach offers a unique perspective on the scale and nature of drug use in modern societies [8,9]. Pharmaceuticals, drugs, and their metabolites have been progressively detected on various denominations of banknotes and on payment cards [8,10,11,12,13,14,15,16,17,18,19,20].

Numerous studies have identified contamination on commonly circulated currencies such as British pounds, euros, and US dollars, with cocaine being the most prevalent substance detected on these banknotes [8,10,11]. Drugs can contaminate banknotes during handling, originating either from dealers or users. Contamination can also occur through direct contact between banknotes, such as during payment transactions or ATM withdrawals. The highest recorded drug contamination on a banknote in the Central European region, encompassing Slovakia, Czech Republic, and Austria, is approximately 1940 ng of cocaine. However, banknotes from festivals have shown significantly higher contamination levels, particularly with methamphetamine, where the maximum observed was about 4660 ng per banknote [9].

The discovery that banknotes, especially those frequently used in transactions like British pounds, euros, and US dollars, are often contaminated with cocaine was not entirely unexpected. This is because these notes can come into direct contact with drugs during transactions. Moreover, it is common for users to roll up a banknote for snorting cocaine, further contributing to the contamination [21,22]. Traces of drugs have been found not only on banknotes and payment cards but also on devices like banknote readers, photocopiers, and printers, which are easily accessible to the public. Banknote circulation can potentially facilitate the global spread of contaminants [21,23].

This study aimed to evaluate the potential and extent of drug and pharmaceutical contaminant transfer from banknotes and credit cards to humans. It also sought to assess how these contaminants contribute to the total micropollutants found in wastewater. Our research concentrated on detecting both illegal and legal drugs, including their metabolites, on the hands of cashiers at a multinational woodworking and furniture company, as well as among the general Slovak population. This study provides the first comprehensive characterization of pharmaceuticals and illicit drugs transferred from banknotes onto humans in Slovakia, significantly expanding current understanding of occupational exposure risks. Moreover, our manuscript brings a novel approach and risk assessment on the integration of occupational health considerations, micropollutant exposure assessment, and environmental contamination pathways in the context of daily handling of currency. Finally, the results of our study could have implications for occupational safety guidelines, hygiene practices, and environmental regulations, suggesting practical strategies for reducing micropollutant exposure and environmental contamination. These results are linked to our broader study on the contamination of circulating banknotes in the Central European region.

## 2. Materials and Methods

Throughout this study, 148 pharmaceuticals, both legal and illegal drugs, and their metabolites were analyzed. The native and isotope-labeled compounds used for preparing the stock solution in methanol (LC-MS grade, Sigma-Aldrich, Prague, Czech Republic) are listed in Table S1. Acetonitrile and formic acid (both LC-MS grade) used as mobile phase components were all obtained from Sigma-Aldrich. Ultra-pure water, used as part of the mobile phase, was prepared with an AquaMax Basic 360 Series and Ultra 370 Series instrument (Young Lin Instrument, Anyang, Republic of Korea). The selection of 148 pharmaceuticals and illicit drugs was based on usage frequency: Pharmaceuticals regularly prescribed in Slovakia and Europe (antibiotics, analgesics, beta-blockers, antidepressants, etc.) were prioritized; environmental relevance: Compounds frequently detected in wastewater, surface waters, and human biomonitoring studies according to literature reports; and health and forensic significance: Substances known to pose public health risks or that are relevant to forensic investigations (e.g., cocaine, methamphetamine, MDMA) were included.

The investigation included examining hand contamination through two volunteer groups. The first group in this study comprised cashiers from a multinational woodworking and furniture company, assumed to have higher contamination levels than the second group. This Group I included 10 cashiers who completed an 8-h work shift. Company statistics indicate that, on average, 5000 customers make payments at the tills on a standard working day, meaning each cashier typically serves about 500 customers per shift. Notably, approximately 35% of these transactions were made with bank cards, which could also carry drug or pharmaceutical residues from daily handling [9]. This scenario suggests significant potential for the transmission of drugs and pharmaceuticals to cashiers through both banknotes and cards. Initially, they thoroughly washed their hands with detergent to eliminate any pre-existing contaminants. Throughout their shifts, they avoided washing or wiping their hands.

Ten volunteers from comparative control Group II, consisting of primarily scientific institution members (PhD students (3), teachers (2), technical service employees (1), students (2), and researchers (2)), participated in the experiment. The sample of volunteers did not contain any individuals employed as cashiers during the experiment. The transfer of micropollutants from the banknotes to the hands of the volunteers was facilitated by rubbing and scratching the banknotes against the fingers and palms of the hands [9].

Hand-Washing Procedure: Participants washed each hand separately by gently immersing one hand in 500 mL of ultra-pure water and rinsing for 30 s, then repeating the process with the other hand using water with detergent. Each rinse was conducted independently, ensuring no cross-contamination occurred between participants and rinsing solutions.

Prevention of Cross-Contamination: To avoid external contamination, each volunteer performed handwashing individually under controlled laboratory conditions, supervised by researchers. Volunteers rinsed their hands above containers to prevent other materials from entering the sample. All containers and utensils were previously cleaned and rinsed with methanol and ultra-pure water. Procedural blanks were analyzed alongside samples to ensure no background contamination.

Both study groups (cashiers and a sample of the general community) provided samples, which were subsequently transported to the laboratory at −4 °C in a freezer box. All collected samples were stored at −20 °C, thawed at room temperature prior to analysis, homogenized, and filtered through a 0.20 μm pore regenerated cellulose syringe filter (Labicom, Olomouc, Czech Republic). Isotopically labeled internal standards were added to the filtered samples (10 mL). Sample analysis was performed using in-line solid phase extraction followed by liquid chromatography coupled with tandem mass spectrometry (in-line SPE-LC-MS/MS). LC-MS/MS analyses were performed using a TSQ Quantiva triple quadrupole mass spectrometer with heated electrospray in both ionization mode coupled with an Accela 1250 pump (both Thermo Fisher Scientific, Waltham, MA, USA) and an HTS XT-CTC autosampler (CTC Analytics AG, Zwingen, Switzerland). LC-MS instrument was controlled by Thermo Scientific Xcalibur 4.1 software (Thermo Fisher). A 1 mL aliquot of each water sample was injected onto an SPE column (Hypersil Gold aQ, 20 mm × 2.1 mm, 12 μm particles), and the retained analytes were subsequently eluted onto an analytical column (Hypersil Gold aQ, 50 mm × 2.1 mm, 5 μm particles) using gradient elution (Table S2). The mobile phases consisted of water (A) and acetonitrile (B), both acidified with 0.1% formic acid. Reported concentrations of pharmaceuticals and illicit drugs represent the average of triplicate measurements. A detailed description of the HPLC-MS/MS method, along with its performance and validation parameters, has been previously published [24,25,26,27]. The concentration of target analytes was calculated based on a combination of internal standards addition and matrix matching standard methods [26,28].

The analysis was conducted on each sample three times. Each analyte’s final value is the average of the data collected from each sample. The instruments utilized have a relative standard deviation of measurement ranging from 15% to 20% [29].

The amounts of each compound adsorbed per person per day (ng/day/person) were calculated using the following formula:

Adsorbed compound (ng/day/person) = C × V(1)

where C is the concentration of a compound detected in the hand rinse water (ng/L) and V is the volume of water used for rinsing (0.5 L per handwashing event, representing typical handwashing practice after an 8-h shift). Thus, values provided reflect the daily potential transfer of micropollutants from banknotes to individuals based on realistic handwashing scenarios.

## 3. Results

Table 1 and Table 2 summarize the drugs, pharmaceuticals, and their metabolites analyzed in samples from Groups I and II, showing average concentrations per volunteer. The findings indicate that micropollutants can transfer from banknotes and credit cards to humans, continuing into wastewater post-handwashing. Despite the potential for contamination from individual handling, credit and debit cards are considered to play a minimal role as a direct vector for micropollutant transfer to cashiers, particularly in the context of the predominance of contactless payments. Consequently, banknotes persist as the predominant focal point with regard to micropollutant exposure risks within occupational contexts.

The data in Table 1 and Table 2 represent the concentration of micropollutants measured on the hands of Groups I and II, respectively, and the respective recalculation by converting the volume of water (0.5 L) used to clean the hands and the number of respondents interviewed (10).

Notably, the drug levels in cashiers (Group I) were up to 30 times higher, with erythromycin reaching 65 times the concentration compared to Group II. The presence of cotinine, a metabolite of nicotine, underscores the significant number of smokers in the Slovak population [7,30]. This study found that cocaine and methamphetamine had the highest concentrations among illicit drugs. In Group II, cocaine was present but at concentrations up to 14.7 times lower than in Group I, highlighting cocaine as a notably expensive drug in Slovakia [7].

Therefore, its presence is likely more common on higher denomination banknotes. The higher concentrations of cocaine in Group I samples can be attributed to the frequent use of higher denomination banknotes (50, 100, and 200 euros) for transactions. This group also showed a significant presence of cocaine’s dominant metabolite, benzoylecgonine. Conversely, methamphetamine, known for its lower cost, emerges as the drug of choice in Slovakia, a fact underscored by studies on its detection in both sewage and circulating banknotes within the country [7,9].

Of the pharmaceuticals, the antibiotics erythromycin and azithromycin, which are used for inflammation and diseases such as urinary tract infections, were identified in the highest concentrations in Group I [29]. In Group II, the analgesic diclofenac and the antibiotic penicillin V were identified. The higher antibiotic concentrations can be justified by the period in which this research was conducted. In November, a long-term increase in inflammatory diseases can be observed in Slovakia, where erythromycin, penicillin V, ciprofloxacin, azithromycin, and clarithromycin are among the most prescribed antibiotics [29]. Commonly used painkillers include diclofenac and tramadol. Higher concentrations of diclofenac were measured in both groups. In Group I, an increased prevalence of *β*-blockers, especially bisoprolol, was also observed. This group of drugs is preferentially prescribed for cardiovascular diseases [29].

## 4. Discussion

The results of this study provide significant insights into the contamination of banknotes with pharmaceuticals and illicit drugs, with particular emphasis on the implications for public health, occupational exposure, and environmental pollution. By comparing these findings to existing literature, a more comprehensive understanding of the dynamics and implications of drug and pharmaceutical contamination on banknotes emerges. Previous research has consistently demonstrated the pervasive contamination of banknotes with trace amounts of drugs and pharmaceuticals [9,10,11,12,13,14,18,20,21,31]. Studies by Amaral et al. (2022) [8] and Pinorini et al. (2020) [12] revealed widespread contamination of both paper and polymer banknotes, with cocaine being the most frequently detected substance [8,10,12]. Similarly, Armenta and de la Guardia (2008 [10]) highlighted that drug residues adhere to banknotes through direct contact during transactions or intentional use, such as rolling banknotes for drug consumption [10]. These findings are corroborated by this study, which identified significantly higher levels of cocaine on the hands of cashiers (Group I) compared to the general population (Group II). The elevated levels of benzoylecgonine, a cocaine metabolite, further support the hypothesis of direct and repeated exposure.

Interestingly, methamphetamine emerged as another prominent contaminant, particularly on banknotes obtained from festivals, as noted in several Central European studies. This aligns with findings from Mackuľak et al. (2016) [9], who reported methamphetamine as a prevalent drug on Slovak banknotes [9]. This study expands on this knowledge by emphasizing the occupational risks for cashiers, who exhibited fivefold higher methamphetamine levels compared to non-cashiers. The type of banknote material plays a critical role in drug retention and transfer. Amaral et al. (2022) [8] demonstrated that polymer banknotes exhibit lower retention rates for cocaine compared to paper banknotes, yet their non-porous surfaces facilitate higher transfer rates to human skin [8]. This finding is crucial for interpreting the contamination levels observed in this study. Given the transition of several countries to polymer banknotes, this could have long-term implications for contamination dynamics and exposure levels.

Moreover, the frequent handling of banknotes by cashiers, coupled with the lack of handwashing during shifts, exacerbates their exposure. This study’s methodology, involving controlled rubbing of banknotes, mirrors real-world scenarios and underscores the role of occupational exposure in elevating contamination levels. This is further supported by the findings of Ebejer et al. (2007) [17], who noted that socio-economic factors and transaction frequencies significantly influence contamination patterns [17]. The significant presence of antibiotics, such as erythromycin and azithromycin, on banknotes highlights an emerging public health concern. Elevated detection rates of these pharmaceuticals in Group I reflect both seasonal trends in antibiotic use and the potential for environmental dissemination through wastewater. Our previous study has documented similar patterns, highlighting the role of wastewater treatment plants (WWTPs) as conduits for micropollutants [30]. The observed antibiotic concentrations in this study align with these findings, emphasizing the dual impact of occupational exposure and subsequent environmental contamination [6,22,30,32,33,34].

The presence of nicotine metabolite cotinine further underscores the interaction between societal behaviors and banknote contamination. As noted by Troiano et al. (2017) [11], cotinine levels serve as biomarkers for population-wide smoking habits, suggesting that banknotes may act as repositories for other societal-level exposures [11]. The release of biologically persistent pharmaceuticals and drugs into wastewater during handwashing represents a secondary contamination pathway. Our study’s findings, indicating significant contributions of carbamazepine, diclofenac, and tramadol to WWTP influent, echo prior research on micropollutants’ persistence and ecological impact. The degradation-resistant nature of these compounds amplifies their environmental footprint, with potential ramifications for aquatic ecosystems [6,22,30].

Additionally, the observed differences in contaminant transfer between detergent and water-only washing highlight the role of surfactants in mobilizing hydrophobic substances. This aligns with findings by Sleeman et al. (2000) [19], who demonstrated enhanced recovery rates of cocaine through solvent extraction techniques [19]. The forensic utility of banknote contamination analysis extends beyond public health surveillance. Pinorini et al. (2020) [12] and Wimmer and Schneider (2011) [18] emphasized the evidentiary value of drug residues on banknotes in criminal investigations [12,18]. This study supports such applications, particularly in distinguishing “dirty money” from innocuously contaminated notes. However, as highlighted by Sleeman et al. (2000) [19], statistical thresholds and regional background levels must be established to strengthen the forensic validity of such evidence [19].

While this study provides valuable insights, several limitations must be acknowledged. The sample size, particularly for Group I, limits the generalizability of the findings. Additionally, the exclusive focus on Slovak banknotes necessitates caution when extrapolating results to other regions with differing contamination dynamics. Future studies should explore longitudinal trends in banknote contamination, incorporating diverse geographical and socio-economic contexts.

Moreover, the integration of advanced analytical techniques, such as Raman spectroscopy and ultra-high-performance liquid chromatography (UHPLC), could enhance the detection and quantification of a broader range of contaminants [10,18]. Investigating the efficacy of alternative banknote materials in reducing contamination transfer could also inform policy decisions on currency design. This study underscores the multifaceted implications of banknote contamination with pharmaceuticals and illicit drugs. By bridging occupational exposure, public health, and environmental pollution, it highlights the interconnected nature of these issues. The findings reinforce the need for targeted interventions, including improved hygiene practices, enhanced wastewater management, and robust forensic protocols. Such measures are essential to mitigate the risks posed by contaminated banknotes, ensuring both public health and environmental integrity.

Our results also indicate that the maximum percentage contribution to the total micropollutants in the WWTP influent can be attributed to biologically inert drugs or psychoactive substances. The contribution of these compounds is at most a millionth of a percent of the mass concentration arriving in the effluent at the WWTP. However, it is important to recognize that every single resident in the city contributes to pollution, as they use banknotes or payment cards daily. It should also be noted that the percentages are theoretical in nature, as some of the micropollutants identified may undergo biological and chemical degradation processes or sorption in the sewage system before reaching the treatment plant. For example, penicillin-V, detected in Groups I and II (directly after hand washing) at higher levels than at the WWTP influent, is degraded and hydrolyzed directly in the sewage system and is present only in negligible quantities at the WWTP influent. In contrast, for pharmaceuticals and drugs that are biologically and chemically inert, such as carbamazepine, diclofenac, or tramadol, this contribution holds interest.

### Assessment of Potential Health Risks

It is of particular concern that occupational exposure to micropollutants via the daily handling of contaminated banknotes may present chronic health risks. This exposure is of particular concern in relation to antibiotics such as erythromycin and azithromycin, as well as illicit drugs like cocaine and methamphetamine, which have been detected on banknotes in circulation. While the concentrations detected harmful compounds that are relatively low, the repetitive and prolonged nature of the exposure has the potential to generate cumulative effects especially among susceptible populations. Furthermore, the presence of some substances increases, additionally, the concerns about broader public health issues such as the potential development of antibiotic resistance and the accident spread of some controlled substances. There is a clear need for further detailed toxicological studies and comprehensive risk assessments that focus on the occupational health standards applicable to cash-handling employees. The aim of such research should be to identify the specific pathways through which these contaminants are absorbed, determine the threshold levels that may cause adverse health effects, especially in sensitive people, and propose guidelines to mitigate exposure risks. Similarly, monitoring and standardization of testing protocols are essential for the health of workers regularly exposed to such micropollutants, thereby contributing to improved occupational safety and public health outcomes.

## 5. Conclusions

This study explores the potential for monitoring the presence of 148 selected drugs, pharmaceuticals, and their metabolites on the hands of cashiers who regularly handle banknotes in circulation across Slovakia, comparing the results with the general population. The findings reveal that pharmaceuticals, particularly antibiotics, can significantly contaminate cashiers, especially during the winter months when this study was conducted. For instance, azithromycin was detected 23 times more frequently and erythromycin up to 64 times more often on the hands of cashiers compared to the general population sample.

The above study confirms that banknotes are not only a medium of exchange for goods and services, but also an important vector for the transmission of legal and illegal micropollutants. The significantly higher concentrations of antibiotics and illicit drugs on the hands of cashiers compared to the general population indicate that professionals who handle cash daily are subject to increased exposure. A notable increase in the prevalence of illicit drugs was also observed, with methamphetamine levels rising fivefold and cocaine up to fifteenfold. The results suggest that hand washing by the general population may contribute to wastewater contamination, representing a secondary pathway through which biologically persistent pharmaceuticals and drugs—such as carbamazepine, diclofenac, and tramadol—enter the environment via rinsing and wastewater discharge.

These findings may stimulate further research and provide practical recommendations for hygiene and public health. At the same time, there is scope for increased collaboration between environmental analysts, pharmacists, and forensic specialists to gain a deeper insight into the dynamics of micropollutant distribution, to improve methods for removing them from wastewater, and to minimize the health and societal risks associated with their presence in circulation.

## Figures and Tables

**Table 1:** Prevalence of illicit drugs and metabolites in Group I and II and subsequent conversion to ng of compound per day per person.

Compound	Group I		Group II	
	ng L^−1^	ng d^−1^ Person^−1^	ng L^−1^	ng d^−1^ Person^−1^
Cotinine	39,000	1900	1400	68
Cocaine	560	28	38	1.9
Methamphetamine	240	12	58	2.9
Benzoylecgonine	130	6.4	16	0.8
Tramadol	27	1.4	8.4	0.4
MDMA	19	1.0	<4.9	-
Citalopram	16	0.8	<1.9	-
Amphetamine	15	0.8	<3.8	-
Methadone	11	0.6	<2.4	-
Oxazepam	2.5	0.1	<2.2	-
THC-COOH	<2	-	17	0.9

**Table 2:** Prevalence of pharmaceuticals and metabolites in Group I and II and subsequent conversion to ng of compound per day per person.

Compound	Group I		Group II	
	ng L^−1^	ng d^−1^ Person^−1^	ng L^−1^	ng d^−1^ Person^−1^
Erythromycin	1600	78	54	2.7
Bisoprolol	960	48.1	<3	-
Azithromycin	820	41	<28	-
Penicillin-V	310	16	320	16
Diclofenac	120	6.0	560	28.0
Ciprofloxacin	89	4.5	41	2.1
Metoprolol	52	2.6	<17	-
Sulconazole	45	2.3	2.6	0.1
Sulfapyridine	24	1.2	<6	-
Carbamazepine	23	1.2	210	10.4
Clarithromycin	22	1.1	<17	-
Fluconazole	20	1.0	<4.5	-
Itraconazole	20	1.0	<18	-
Venlafaxine	20	1.0	<3	-
Clotrimazole	18	0.9	11	0.6
Terbutaline	18	0.9	<5	-
Atenolol	16	0.8	13	0.7
Levofloxacin	12	0.6	<5.9	-
Sulfamethazine	12	0.6	<6	-
Sulfamethizole	12	0.6	<6	-
Mirtazapine	<9.7	-	6.0	0.3
Codeine	9.5	0.5	6.2	0.3
Promethazine	9.5	0.5	<3.2	-
Valsartan	8.4	0.4	4.7	0.2
Isradipine	7.7	0.4	<4.3	-
Telmisartan	<7.1	-	10	0.5
Ranitidine	4.0	0.2	<0.9	-
Terconazole	4	0.2	<2.1	-
Verapamil	3.6	0.2	<2.8	-
Clonazepam	1.2	0.1	<0.76	-
Diphenhydramine	0.6	0.0	<0.36	-

## Data Availability

All data supporting the findings of this study are available within this article and the Supplementary Materials. Additional raw data and analytical method validation details are available from the corresponding authors upon reasonable request.

## Supplementary Materials

The following supporting information can be downloaded at: https://www.mdpi.com/article/10.3390/toxics13040242/s1, Table S1: Target compounds, internal standards (IS), MS transitions, used for LC-MS analysis; Table S2: The gradient for the elution of target compounds for in-line SPE (A) and LC (B).
