# Supplementary material for: Banknotes as a Source of Drug and Pharmaceutical Contamination of the Population
Source: Toxics. 2025 Mar 24;13(4):242. doi: 10.3390/toxics13040242 (PMC12031460; doi:10.3390/toxics13040242)
Supplement: Supplementary file 1 [file toxics-13-00242-s001.zip › toxics-3458637-supplementary.pdf]

SM1: Target compounds, internal standards (IS), MS transitions, used for LC-MS analysis.

Analyte	Group	Mode	Precursor ion	Quan	Qual	CE [V]	RF Lens [V]	RT [min]
2-oxo-3-hydroxy-LSD	illicit drugs	+	356.2	237.1	222.1	24/32	69	5.9
6- acetylmorphine	illicit drugs	+	328.2	165.1	211.1	38/26	85	5.6
6- acetylmorphine _IS		+	334.3	165.1		38	79	5.6
Alfuzosin	alpha blockers	+	390.2	235.0	156.1	27/27	93	6.6
Alprazolam	benzodiazepines	+	309.1	205.0	281.0	42/26	89	8.9
Amiodarone	antiarrhythmic agents	+	646.0	645.2	99.9	12/27	137	10.7
Amitriptyline	antidepressants	+	278.2	233.1	191.1	26/18	63	9.9
Amitriptyline_IS		+	284.2	233.1		18	64	9.9
Amphetamine	illicit drugs	+	136.1	91.1	119.1	19/10	30	5.3
Amphetamine_IS		+	141.1	93.1		10	30	5.3
Atenolol	beta blockers	+	267.2	145.1	190.1	27/18	63	4.8
Atenolol_IS		+	273.3	145.1		27	63	4.8
Atorvastatin	statins	+	559.3	440.2	466.2	20/14	86	9.8
Atracurium	muscle relaxants	+	358.2	205.7	150.8	18/28	106	5.7
Azithromycin	antibiotics	+	749.5	591.5	573.4	28/33	104	6.9
Azithromycin_IS		+	752.7	576.4		28	112	6.9
Benzoylecgonine	illicit drugs	+	290.2	168.1	105.1	19/30	62	6.0
Benzoylecgonine_IS		+	298.2	171.1		20	60	6.0
Bezafibrate	lipid-lowering agents	+	362.1	139.0	121.0	26/29	59	9.3
Biperiden	antiparkinsonian agents	+	312.3	98.1	294.1	23/16	60	9.4
Bisoprolol	beta blockers	+	326.2	116.1	74.1	17/26	72	7.6
Budenoside	glucocorticoid steroids	+	431.2	146.9	172.7	32/25	108	8.3
Buprenorphine	semi-synthetic opioids	+	468.3	413.8	467.6	31/13	154	7.7
Caffeine	stimulant	+	195.2	138.0	110.1	20/24	62	5.2
Caffeine_IS		+	198.1	140.1		20	62	5.2
Cannabinol	illicit drugs	+	311.2	223.1	208.1	21/31	65	7.0
Carbamazepine	anticonvulsants	+	237.1	194.1	192.1	20/25	61	8.0
Carbamazepine_IS		+	247.2	204.1		22	64	8.0
Cetirizine	antihistamines	+	389.2	201.0	165.1	18/55	57	9.5
Chloramphenicol	antibiotics	-	320.9	151.9	150.9	18/22	90	4.0

Chlorpromazine	psycholeptics	+	319.1	213.7	86.0	18/39	102	9.3
Chlorprothixene	psycholeptics	+	316.1	230.8	220.9	27/37	101	9.5
Cilazapril	antihypertensions	+	418.2	211.1	70.1	18/41	59	9.1
Ciprofloxacin	antibiotics	+	332.1	287.7	230.7	14/30	103	5.7
Citalopram	antidepressants	+	325.2	109.1	262.1	27/19	72	8.9
Citalopram_IS		+	331.3	262.1		20	80	8.9
Clarithromycin	antibiotics	+	748.5	590.3	558.4	17/21	83	8.8
Clarithromycin_IS		+	751.5	593.4		17	84	8.8
Clemastine	antihistamines	+	344.3	215.0	130.2	17/10	45	11.0
Clindamycin	antibiotics	+	425.2	126.1	377.1	28/18	75	7.4
Clindamycin_IS		+	428.2	129.2		28	75	7.4
Clindamycin sulfoxide	active metabolite	+	441.1	377.1	126.2	16/30	61	6.4
Clomipramine	antidepressants	+	315.2	86.1	227.1	18/42	58	10.5
Clonazepam	anticonvulsants	+	316.1	214.0	270.0	38/38	83	8.6
Clotrimazole	antifungal medications	+	277.1	165.1	241.1	23/26	68	9.2
Cocaine	illicit drugs	+	304.2	182.1	150.1	20/25	63	7.3
Cocaine_IS		+	307.2	85.2		28	62	7.3
Codeine	opiate analgesics	+	300.2	165.1	215.1	42/25	75	5.2
Cotinine	alkaloids	+	177.2	98.1	146.0	20/17	68	5.8
Desloratadine	antihistamines	+	311.1	258.7	257.7	18/32	100	6.2
Diclofenac	NSAID	+	296.0	214.0	215.0	35/19	48	9.9
Diclofenac IS		+	302.0	220.1		33	50	9.9
Dicycloverine	anticholinergics	+	310.3	165.1	237.1	19/20	61	10.5
Difloxacin	antibiotics	+	400.0	298.6	355.7	26/16	118	6.7
Dihydroergotamine	antimigraine agents	+	584.3	269.7	252.7	26/30	142	7.4
Diltiazem	calcium channel blockers	+	415.2	178.0	150.1	24/42	74	9.0
Diphenhydramine	antihistamines	+	256.2	167.9	165.1	13/40	35	8.8
Disopyramide	antiarrhythmic agents	+	340.2	239.1	195.1	17/29	58	7.1
Donepezil	anti-Alzheimer drugs	+	380.3	91.0	243.1	36/26	83	8.3
Duloxetine	antidepressants	+	298.1	182.7	44.1	19/13	66	8.5
Econazole	antifungal medications	+	381.0	124.8	192.6	29/16	102	10.0
Enoxacin	antibiotics	+	321.1	233.7	205.6	20/26	73	5.3
Enrofloxacin	antibiotics	+	360.2	316.1	245.0	16/22	76	6.1
Eprosartan	antihypertensive drugs	+	425.2	206.8	134.8	22/30	114	7.3

Erythromycin	antibiotics	+	734.5	576.4	558.4	18/17	80	8.5
Felodipine	calcium channel blockers	+	384.1	305.8	323.7	21/19	89	10.5
Fenofibrate	antihyperlipidaemics	+	361.2	233.0	139.0	16/28	59	11.1
Fexofenadine	antihistamines	+	502.3	466.2	484.3	26/22	89	9.7
Fexofenadine_IS		+	508.3	472.3		26	102	9.7
Finasteride	urological	+	373.3	304.9	372.5	27/5	117	8.8
Flecainide	antiarrhythmic agents	+	415.1	397.9	300.6	21/30	113	7.9
Florfenicol	antibiotics	-	356.1	183.9	336.0	19/10	63	3.3
Fluconazole	antifungal medications	+	306.8	237.5	219.5	14/18	100	5.7
Flumequine	antibiotics	+	262.1	201.6	243.7	31/18	94	7.6
Flutamide	hormone-antagonists	+	275.0	202.0	205.0	25/23	100	8.8
Fulvestrant	hormone-antagonists	+	607.3	588.9	492.6	21/13	111	9.9
Furosemide	diuretics	+	328.7	285.0	205.0	17/24	89	7.0
Glibenclamide	antidiabetic drugs	+	494.2	369.0	169.0	13/34	57	9.9
Glimepiride	antidiabetic drugs	+	491.3	352.1	126.1	12/25	57	10.1
Haloperidol	antipsychotic drugs	+	376.1	165.1	123.1	23/38	76	9.3
Hydroxyzine	antihistamines	+	375.2	165.5	200.6	35/16	92	8.40
Iopromide	iodinated contrast media	+	791.8	572.8	558.8	26/31	109	4.6
Indometacin	NSAID	+	358.0	138.7	110.7	20/44	4	5.6
Irbesartan	angiotensin II receptor blockers	+	429.3	207.1	195.1	23/22	73	9.2
Irbesartan_IS		+	433.2	195.3		24	83	9.2
Ketamine	NMDA receptor antagonists	+	238.1	125.1	207.1	28/14	50	6.2
Isradipine	calcium channel blockers	+	372.2	311.7	208.4	7/26	76	10.1
Itraconazole	antifungal medications	+	705.2	431.8	704.4	29/16	158	10.6
Ketoprofen	NSAID	+	255.1	208.8	104.8	11/20	89	3.9
Lamotrigine	antiepileptic	+	256.0	159.0	210.9	29/27	85	6.7
Levofloxacin	antibiotics	+	362.1	260.6	317.6	25/16	74	5.6
Levomepromazine	psycholeptics	+	329.1	100.2	166.7	17/56	92	9.1
Lomefloxacin	antibiotics	+	352.1	264.7	236.8	22/30	75	5.8
Loperamide	antidiarrheals	+	477.3	266.1	210.1	24/48	78	10.3
Maprotiline	antidepressants	+	278.2	250.1	191.1	18/36	61	9.8
MDA	illicit drugs	+	181.1	164.1	134.1	10/18	30	5.6
MDA_IS		+	185.1	168.1		10	30	5.6

MDEA	illicit drugs	+	208.1	163.1	105.1	14/25	44	6.2
MDMA	illicit drugs	+	194.1	105.1	163.1	24/13	39	5.8
MDMA_IS		+	199.1	165.1		13	42	5.8
Meclozine	antihistamines	+	391.3	201.0	166.1	17/38	55	9.5
Memantine	anti-Alzheimer drugs	+	180.1	163.1	107.1	16/26	30	7.6
Mephedrone	illicit drugs	+	178.1	160.1	145.1	13/21	42	6.1
Methamphemtamine	illicit drugs	+	150.1	91.1	119.1	21/10	40	5.6
Methamphetamine_IS		+	155.2	92.1		21	40	5.6
Methadone	illicit drugs		310.2	265.1	105.1	14/26	53	10.0
Methadone_IS			319.3	268.1		15	57	10.0
Metoprolol	antihypertensive drugs	+	268.2	116.1	191.1	18/18	63	6.7
Metoprolol_IS		+	275.2	123.2		20	54	6.7
Metoprolol acid	metabolites	+	268.2	145.1	190.9	24/16	64	5.5
Mianserin	antidepressants	+	265.2	208.1	263.1	21/20	64	8.8
Miconazole	antifungal medications	+	417.2	159.0	161.0	30/31	91	11.5
Mirtazapine	antidepressants	+	266.2	195.1	209.1	26/20	61	6.4
Morphine	illicit drugs	+	286.1	152.1	165.1	55/40	77	4.3
Naproxen	NSAID	-	229.1	169.0	141.0	36/53	77	4.4
Nefazodone	antidepressants	+	470.2	273.7	245.6	25/30	120	9.2
Norfloxacin	antibiotics	+	320.1	275.5	233.1	13/21	102	5.5
Norketamine	metabolites	+	224.1	125.1	207.0	25/10	44	6
Norsertraline	metabolites	+	275.1	159.0	123.0	20/43	57	10.2
Orphenadrine	anticholinergic drugs	+	270.2	181.1	270.2	12/29	35	8.4
Oseltamivir carboxylate	antivirals	+	285.2	138.1	180.1	19/10	42	5.5
Oxazepam	psycholeptics	+	287.0	241.0	269.0	23/14	65	8.2
Oxazepam_IS		+	292.1	246.1		23	65	8.2
Oxycodone	opioid analgesics	+	300.2	165.1	215.0	42/25	75	4.9
Oxytetracycline	antibiotics	+	461.1	426.0	443.1	17/10	66	5.3
Paroxetine	antidepressants	+	330.1	191.8	329.4	17/5	76	8.2
Penicillin-V	antibiotics	-	349.2	208.0	305.1	10/10	46	6.7
Pizotifen	antimigraine agents	+	296.2	96.1	199.1	23/26	72	8.7
Promethazine	antihistamines	+	285.2	86.0	197.6	15/27	97	8.4
Propranolol	beta-blockers	+	260.2	116.1	183.1	18/18	60	7.5
Ranitidine	antihistamines	+	315.1	130.1	176.0	12/24	51	4.6

Risperidone	psycholeptics	+	411.2	190.9	109.8	26/43	113	6.6
Ropinirole	antiparkinsonian agents	+	261.2	114.2	160.1	19/23	62	5.8
Rosuvastatin	statins	+	482.1	258.1	270.1	33/36	101	8.9
Roxithromycin	antibiotics	+	837.5	679.4	522.3	33/36	101	8.8
Sertraline	antidepressants	+	306.0	159.0	275.0	28/10	37	8.07
Sotalol	antihypertensive drugs	+	273.1	255.0	213.1	10/18	48	4.5
Sulconazole	antifungals medications	+	399.0	126.7	182.6	31/14	111	10.1
Sulfadiazine	antibiotics	+	251.1	156.0	108.0	15/24	55	4.6
Sulfamerazine	antibiotics	+	265.2	172.0	156.9	16/17	60	5.0
Sulfamethazine	antibiotics	+	279.1	186.0	124.1	17/24	64	5.3
Sulfamethizole	antibiotics	+	271.1	156.0	108.0	14/24	52	5.6
Sulfamethoxazole	antibiotics	+	254.1	156.0	108.0	16/24	55	6.3
Sulfamethoxazole_IS		+	260.0	162.0		16	55	6.5
Sulfadimethoxine	antibiotics	+	311.1	155.6	107.8	17/25	70	6.3
Sulfamethoxypyridazine	antibiotics	+	281.0	157.7	107.6	14/23	67	4.5
Sulfamoxol	antibiotics	+	268.0	155.6	107.8	14/23	59	4.1
Sulfaphenazole	antibiotics	+	315.1	158.2	130.8	26/41	71	6.5
Sulfapyridine	antibiotics	+	250.2	156.0	184.1	16/18	59	4.9
Sulfaquinoxaline	antibiotics	+	301.0	155.7	107.7	14/24	64	6.4
Sulfasalazine	antibiotics	+	399.1	380.1	222.6	15/28	87	6.9
Sulfathiazole	antibiotics	+	256.0	155.7	91.7	11/25	93	3.7
Tamoxifen	hormone-antagonists	+	372.3	72.1	129.1	24/27	78	10.7
Telmisartan	angiotensin II receptor blocker	+	515.3	497.2	276.0	33/46	127	8.8
Telmisartan_IS		+	519.3	280.1		48	127	8.8
Terbinafine	antifungal medications	+	292.2	141.1	115.0	22/55	54	9.7
Terbutaline	β2-adrenergic receptor agonist	+	226.1	152.1	125.1	16/24	46	4.5
Terconazole	antifungal medications	+	532.2	219.1	277.1	30/26	99	10.6
Tetracycline	antibiotics	+	445.1	153.7	409.7	24/18	64	5.7
THC-COOH	Illicit drugs		345.3	327.1	299.2	15/19	64	10.5
THC-COOH_IS			354.3	308.2		20	64	10.5
Theophylline	xanthines	-	179.0	164.0	179.0	20/20	67	4.7

Tramadol	analgesics	+	264.2	58.1	264.1	17/10	46	6.3
Tramadol_IS		+	267.2	58.1		30	46	6.3
Trazodone	antidepressants	+	372.2	148.0	176.0	33/24	81	7.3
Triamterene	potassium-sparing diuretics	+	254.1	237.0	141.0	27/46	96	5.9
Trihexyphenidyl	antiparkinsonian agents	+	302.2	98.2	70.0	20/37	99	8.9
Trimethoprim	antibiotics	+	291.2	230.1	261.1	23/25	78	5.6
Trimethoprim_IS		+	294.2	231.1		23	78	5.6
Valsartan	angiotensin II receptor blockers	+	436.2	291.1	235.1	17/17	52	9.1
Venlafaxine	antidepressants	+	278.2	58.1	260.2	19/10	49	7.1
Venlafaxine_IS		+	284.3	266.2		10	49	7.1
Verapamil	antihypertensive drugs	+	455.4	165.1	303.2	27/25	88	8.8
Vortioxetine	antidepressants	+	299.2	150.1	256.0	24/20	71	9.5

Quan = quantitative ion; Qual = qualitative (confirmation) ion; RT = retention time
All standards were purchased from AK Scientific (Union City, CA USA), Alsa Chim (Illkirch Graffenstaden, France), Cerilliant (Wesel, Germany), Chemos (Altdorf, Germany), Chiron (Trondheim, Norway), Lipomed AG (Arlesheim, Switzerland), Santa Cruz Biot. (Heidelberg, Germany), Sigma Aldrich (Prague, Czech Republic)
All purchased chemicals had analytical grade certificate or purity > 98%;
TRC … Toronto Research Chemicals, EP … European Pharmacopoeia

SM2: The gradient for the elution of target compounds for in-line SPE (A) and LC (B).

(A)

Time [min]	Water + 0.1% FA [%]	Acetonitrile + 0.1% FA [%]	Flow [µL min $^{-1}$]
0.00	100	0	1100
1.05	100	0	1100
1.07	0	100	100
11.10	0	100	50
12.00	0	100	2000
12.10	100	0	1100
15.00	100	0	1100

(B)

Time [min]	Water + 0.1% FA [%]	Acetonitrile + 0.1% FA [%]	Flow [µL min $^{-1}$]
0.00	100	0	300
1.00	100	0	300
7.00	60	40	350
10.00	20	80	400
12.00	0	100	450
13.00	0	100	450
13.01	100	0	300
15.00	100	0	300

FA … formic acid
